# How to do (or not to do)… health resource allocations using constrained mathematical optimization

**DOI:** 10.1093/heapol/czac096

**Published:** 2022-11-18

**Authors:** Robyn M Stuart, Nicole Fraser-Hurt, Zara Shubber, Lung Vu, Nejma Cheik, Cliff C Kerr, David P Wilson

**Affiliations:** Department of Mathematical Sciences, University of Copenhagen, Universitetsparken 5, Copenhagen 2100, Denmark; Burnet Institute, 85 Commercial Road, Melbourne 3004, Australia; The World Bank Group, 2121 Pennsylvania Avenue NW, Washington, DC 20433, USA; The World Bank Group, 2121 Pennsylvania Avenue NW, Washington, DC 20433, USA; The World Bank Group, 2121 Pennsylvania Avenue NW, Washington, DC 20433, USA; The World Bank Group, 2121 Pennsylvania Avenue NW, Washington, DC 20433, USA; Institute for Disease Modeling at the Bill & Melinda Gates Foundation, 500 Fifth Avenue North, Seattle, WA 98109, USA; School of Physics, University of Sydney, Physics Road, Sydney, New South Wales, Camperdown 2006, Australia; Burnet Institute, 85 Commercial Road, Melbourne 3004, Australia; Bill & Melinda Gates Foundation, 500 Fifth Avenue North, Seattle, WA 98109, USA

**Keywords:** Resource allocation, cost-effectiveness analysis

## Abstract

Despite the push towards evidence-based health policy, decisions about how to allocate health resources are all too often made on the basis of political forces or a continuation of the status quo. This results in wastage in health systems and loss of potential population health. However, if health systems are to serve people best, then they must operate efficiently and equitably, and appropriate valuation methods are needed to determine how to do this. With the advances in computing power over the past few decades, advanced mathematical optimization algorithms can now be run on personal computers and can be used to provide comprehensive, evidence-based recommendations for policymakers on how to prioritize health spending considering policy objectives, interactions of interventions, real-world system constraints and budget envelopes. Such methods provide an invaluable complement to traditional or extended cost-effectiveness analyses or league tables. In this paper, we describe how such methods work, how policymakers and programme managers can access them and implement their recommendations and how they have changed health spending in the world to date.

Key messages

- The realities of finite resources mean that health-care packages need to be prioritized. Traditional methods such as cost-effectiveness analysis are one way to do this but have limitations.
- Here we outline how mathematical optimization algorithms can be used to provide comprehensive, evidence-based recommendations for policymakers on how to prioritize health spending and provide an accessible summary of how they work.

## Introduction

As one of the largest components of national budgets, health portfolios receive considerable public and political scrutiny, with plenty of debate around how they should be spent. Whilst many governments and agencies have voiced support for the goal of providing universal health coverage (UHC), it is impossible to consider UHC separately from the realities of finite resources. This means that health-care packages need to be prioritized. Health systems are so complex that, historically, the process of prioritization has often been broken down into discrete, binary decisions. Health interventions are assessed one at a time by comparing the marginal cost of the intervention to the marginal benefits it would deliver—either health-only benefits in a cost-effectiveness analysis (CEA) or also including non-health benefits such as financial risk protection in an extended CEA (Edejer *et al.*, 2003; Chalkidou *et al.*, 2016; Jamison *et al.*, 2018). Intuitively, interventions that deliver the greatest benefits per dollar spent should be implemented first, although the exact criteria that determine whether interventions are in or out are still subject to debate (Marseille *et al.*, 2015; Woods *et al.*, 2016; Glassman *et al.*, 2017; Ochalek *et al.*, 2018).

The problem with assessing health-care interventions one at a time is that they are generally not independent of one another. Even the definition of independence is debated: in a study dedicated to this topic, Dakin and Gray (2018) found ‘substantial variation among 14 published definitions of “independent” in the context of health-care decision-making’.

In any case where interaction effects are present, World Health Organization (WHO) guidelines recommend that the combinations of interventions (e.g. no intervention, A, B and A + B) should be compared incrementally (Edejer *et al.*, 2003), following the methods for a 2 × 2 factorial trial. This has been termed generalized cost-effectiveness analysis or GCEA (Murray *et al.*, 2000). The same guidelines also recommend calculating health gains under different levels of intervention coverage. If we were to follow this advice to compare six interventions, each with five possible coverage levels, there would be more than 15 000 combinations to consider. In view of this complexity and the data required to inform it, it is perhaps not surprising that many studies and advisory bodies ignore interaction effects altogether, even when doing so means missing out on potential health gains (Evans *et al.*, 2006; Dakin and Gray, 2018). The practice of evaluating a proposed new intervention against a no-intervention standard of care scenario is still a pervasive, if not the dominant, method used today (Verguet *et al*., 2021).

But comparing many thousands of scenarios is exactly the kind of task that computers are built for, and optimization algorithms are capable of efficiently estimating the ‘best’ alternative when there are a large number of variables and potential constraints on the solutions (e.g. budget constraints). The task force on Optimization Methods Emerging Good Practices established by the Professional Society for Health Economics and Outcomes Research (ISPOR) published two reports in which they surveyed a range of optimization methods that had been applied to decision-making problems in health and concluded that ‘the application of constrained optimization methods to health-care decision-making offers substantial potential benefits’ (Crown *et al.*, 2017; Crown *et al.*, 2018).

The ISPOR Task Force provides an invaluable introduction to the application of constrained optimization methods to health-care problems and also addresses several of the key barriers that have historically prevented widespread uptake, including data availability and quality and the challenges of model validation, among others. Another noteworthy limitation is that implementing one of these methods typically still requires considerable mathematical and programming abilities and as such can provide a significant barrier to uptake in most policy settings. However, these barriers are increasingly unnecessary: the enormous advances in human–computer interaction over recent years mean that it is now possible to integrate advanced algorithms into easy-to-use software tools, including WebApps, thus empowering people at all levels of technical/mathematical ability to access them.

In this paper, we focus on the question of how to allocate health resources optimally. We start by defining constrained optimization and then discuss how to solve a constrained optimization problem to prioritize health-care interventions. We then discuss when and why such methods are needed. Finally, we discuss how policymakers can access these methods and use them to guide decision-making on health resource allocation.

## What is constrained optimization?

Constrained optimization can be defined as maximizing or minimizing an objective function by changing some variables, subject to some constraints. We can break down this rather broad definition into three components: the objective function, the decision variables and the constraints. Let us consider two motivating examples to illustrate what form these components can take.

### Allocative efficiency among programmes

We first consider an example where the goal is to maximize the health outcomes of a population by varying the allocation of funding, constrained so that the overall spending stays the same. In this case, the objective function is the population health outcomes, the decision variables relate to how this funding should be allocated and the constraint is the overall budget. More precisely, suppose that a country’s health response consists of *n* programmes each with a budget of *B_i_* for *i *= 1,…,*n*. The set of decision variables in this case are the budgets that are allocated to each programme, {*B*_1_,…,*B_n_*}. We also denote the total budget by $T = \mathop \sum \nolimits_{i = 1}^n {B_i}$, i.e. the sum of the budgets to each programme. Mathematically, a constrained optimization problem could be expressed as

$$\max H\left( {{B_1}, \ldots ,{B_n}} \right) s.t.\ T \le C$$

where *H*(${B_1}, \ldots ,{B_n}$) represents the health outcomes under budget allocation {*B*_1_,…,*B_n_*} and *C* is some overall budget constraint. In words, this equation states that we wish to maximize the health outcomes *H*, which will result from a particular budget allocation {*B*_1_,…,*B_n_*}, such that our total budget *T* is equal to or less than our budget constraint *C*.

To illustrate this, let us consider a hypothetical example where we have a total budget of US$1 million (m) to allocate towards type 2 diabetes, which can either be allocated to a screening programme or to a treatment adherence programme. The screening programme is estimated to avert 100 disability-adjusted life years (DALYs), while the treatment adherence programme is estimated to avert 200 DALYs. Using the mathematical notation that we defined above, we have two possibilities: under the first budget allocation {$1 *m*, $0 *m*} we have *H*($1 *m*, $0 *m*) = 100 and under the second we have *H*($0 *m*, $1 *m*) = 200. In this case, funding the treatment adherence programme would be identified as the optimal choice using a constrained optimization framework, and this corresponds to the choice that would be found by a CEA. The problem becomes more complex, however, if we consider options for partially funding each programme; we will explore this more in a later example.

This general form of a constrained optimization can be extended in various ways. For example, it may not be possible to defund an existing programme *a* below its current level $B_a^0$, in which case the problem could be written as

$$\max H\left( {{B_1}, \ldots ,{B_n}} \right)\ s.t.\ T \le C\ and\ {B_a} \ge B_a^0.$$

Alternatively, it may be possible to disaggregate the health outcome among separate subgroups of the population (e.g. children vs adults) and impose a constraint that the health outcome for some population must meet a certain target. For example, suppose that the health outcomes of interest are the number of people with controlled diabetes, and there are specific targets for population *k* (e.g. at least *M* adolescents with controlled diabetes). This can be done by writing

$$\max H\left( {{B_1}, \ldots ,{B_n}} \right)\ s.t.\ T \le C\ and\ {H_k}\left( {{B_1}, \ldots ,{B_n}} \right) \ge M,$$

where ${H_k}\left( {{B_1}, \ldots ,{B_n}} \right)$ are the health outcomes for population *k*.

### Optimal vaccine distribution

For this next example, the goal is to minimize the number of cases of an infectious disease by varying the distribution of vaccines among a population, subject to a constraint on the overall number of doses available. For example, if distributing a vaccine between people over vs under 65, we could write *V* = {*V*_<65_, *V*_65+_} to represent the number of doses allocated to each age cohort and then write the constrained optimization problem as:

$$\min\ infections\left( V \right)\ s.t.\ {V_{ \lt 65}} + {V_{65 + }} \le {V^*},$$

where infections (*V*) represents the total number of infections under vaccine allocation *V* and V* is the total number of doses available. As with the previous example, this can be generalized in various ways; e.g. infections could be replaced by some other objective like deaths.

In both examples presented, the optimal allocation of resources depends in a complex way on the decision variables. For example, targeting influenza vaccines towards people over 65 may avert more deaths, since influenza mortality is higher for those over 65. But on the other hand, if the majority of transmission occurs in people under 65, then having a well-vaccinated adult cohort may prevent secondary infections in the over 65 cohort and thus end up averting more infections.

## A conceptual framework for optimally allocating health resources

### Step 1: clearly identify the problem

The previous section highlighted that constrained optimization consists of an objective function, one or more decision variables and one or more constraints. In practice, the first step in carrying out a constrained optimization is to identify these three components. This is often formulated in plain language, e.g. ‘where should facilities be located to ensure travel times are minimized, whilst also making sure that no-one needs to travel more than 2 hours?’ or ‘how can I arrange my roster to get the most out of my staff, whilst respecting everyone’s availability?’. This step can be deceptively complex; although it is the least technical of the steps to follow, none of the following step can be completed without a clear understanding of the problem at hand. Furthermore, the choice of objective depends on what one considers most important (e.g. minimizing infections or deaths), while the choice of constraints can depend on many things, including financial considerations (such as budget available, although this too might not be fixed, but rather determined in dialogue with other government departments) and equity considerations. This is perhaps the most important step and the one where policymakers are most involved.

### Step 2: determining how outcomes depend on the allocation of resources

If you knew what outcome would result from each possible allocation of resources, then you would be in a good position to determine which allocation was the best. In practice, estimating the outcomes for each budget allocation generally requires data and/or a model. A motivating example is provided in the inset Box 1, where the four feasible allocations are assessed during pilot trials to determine their associated outcomes.

Box 1. **How outcomes depend on the allocation of resources**

Suppose you are the manager of a local health centre and you wish to provide support for osteoarthritis (OA) patients in your district. Currently, OA patients are provided with leaflets containing advice on self-management, as well as four 60-min classes teaching joint protection principles at a total cost of $92/patient. Your overall budget cap is $150/patient. You are considering expanding the classes to 90 min to teach hand exercises as well as joint protection. This will cost additional $30/patient. However, a pilot trial shows that patients struggle to follow both the joint protection and hand exercises, so there is a small decrease in quality-adjusted life years (QALYs) when both are taught in the same class. You therefore decide not to provide hand exercises. However, the occupational therapist in charge of the sessions suggests that she try replacing the joint protection lessons with hand exercises, rather than providing both. She tells you that making this change will decrease the cost/patient by $60 relative to providing both. This time, you find that QALYs are maximized by providing hand exercises alone, compared with providing joint protection classes alone, providing neither joint protection nor hand exercises or providing both.

This example is based on the results reported in a 2015 study (Oppong *et al.*, 2015) that found providing hand exercises without joint protection was the most cost-effective option. However, hand exercises would not appear cost-effective if measured relative to a baseline that included joint protection.

In this example, there are four feasible allocations (hand exercises, joint protection, both or neither), and the outcomes for each allocation are determined by pilot trials. These are summarized below.

- Highest QALYs: provide hand exercises
  ◦ Cost: $32/patient
- Second-highest QALYs: provide joint protection
  ◦ Cost: $92/patient
  ◦ QALYs: medium–high
- Third-highest QALYs: provide both joint protection and hand exercises
  ◦ Cost: $122/patient
  ◦ QALYs: medium
- Baseline (no QALYs): provide neither joint protection nor hand exercises
  ◦ Cost: $0/patient
  ◦ QALYs: none

The decision variables in the problem are which programmes to provide (hand exercises, joint protection, both or neither). The objective is to maximize QALYs. The constraint is the overall budget cap of $150/patient, and since none of the options exceeds this, all four are feasible.

**Figure 1.**
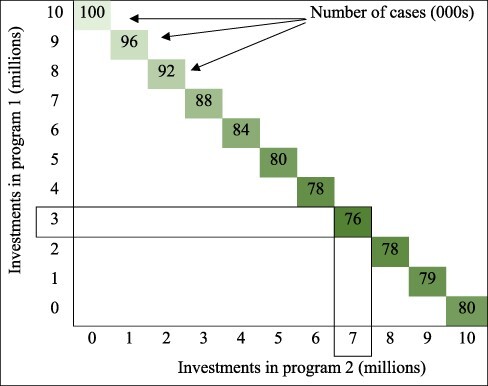
Deciding between two programmes when each one can be implemented at a varying scale

In practice, pilot trials quickly become infeasible when there is a larger or more complex set of feasible allocations, especially if we consider different implementation options for each intervention, such as varying coverage levels or delivery platforms. Instead of a binary choice to either include or exclude each intervention, we now have a continuum of options for each one. Figure 1 illustrates an example where we are choosing between two programmes, each of which cost $10 m at full scale. If Programme 1 is implemented at full scale we project 100 000 cases, and if Programme 2 is implemented at full scale we project 80 000 cases. If the only options are to implement at full scale or not at all, then we would select Programme 2. However, it is possible that partially funding the programmes may deliver greater benefits; for instance, funding Programme 1 at 30% scale may deliver >30% of the associated benefits, particularly if combined with a partial funding of Programme 2. Figure 1 presents an illustrative full budget curve, where we find that the optimal allocation of $10 m is a 30/70 split between Programmes 1 and 2.

For the example provided in Figure 1, it would generally not be possible to evaluate each allocation using pilot trials. Instead, a model could be used to help estimate the outcomes under different allocations.

Our group has worked extensively with resource allocation questions around infectious diseases, for which we have used compartmental disease models combined with costing modules that map spending to model parameters (Kerr *et al*., 2015). Other types of constrained optimization call for different model types; Crown *et al.*, (2017) give an overview of several common types. The fundamental feature of these models is that they formalize the relationship between resource allocations and outcomes.

### Step 3: determining the optimal allocation of resources

Optimization algorithms are designed to search for the optimal solution when there are many options. The use of such algorithms has a long history in health decision sciences (Stinnett and Paltiel, 1996), and there are numerous different types of algorithm that can be applied to different problems, from resource allocation to patient scheduling to facility location planning.

**Figure 2.**
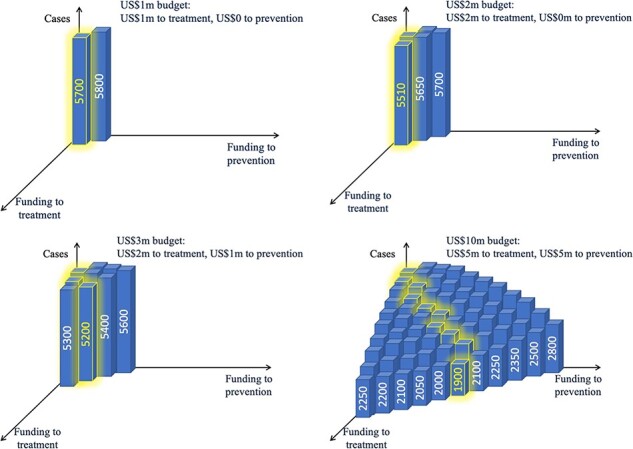
Deciding between preventions and treatment programmes when each one can be implemented at a varying scale, and the optimal allocation between them depends on the total budget available

In Figure 2 we present another example illustrating how optimization algorithms can help determine the optimal allocation of funding between prevention and treatment programmes. The case numbers resulting from each level of funding are made up for the purposes of this example but would typically be calculated using a model and data as outlined in the previous section. In this example, when very little funding (US$1 m) is available, it is better to allocate it all to treatment programmes, which would result in 5700 cases (Figure 2a). With a budget of US$2 m, the best strategy is still to allocate it all to treatment for a total of 5510 cases (Figure 2b), but as the budget increases and treatment programmes are established, it is best to start scaling up prevention programmes—a budget of US$3 m would result in 5300 cases if it was all allocated to treatment, vs 5200 if US$1 m was allocated to prevention programmes (Figure 2c). As the overall budget increases, the number of projected cases decreases, and the optimal allocation between prevention and treatment changes (Figure 2d). There may be several reasons for such non-linear scale effects: there can be a non-linear relationship between the overall expenditure on a programme and programmatic coverage (e.g. if the marginal cost per person covered increases or decreases as the programme scales up), or there can be a non-linear relationship between the magnitude of the programmatic interaction effects as programme coverage increases (e.g. prevention efforts may be more effective if treatment programmes are operating), or there can be non-linear epidemiological effects, especially in the case of infectious diseases, due to herd immunity effects.

To understand how such algorithms work, consider the example presented in Figure 2d. Suppose we initially considered allocating US$10 m to treatment programmes, which would result in 2250 cases. Next, we ask what would happen if we reallocated US$1 m of the total budget to prevention programmes. We calculate that this would lead to 2200 cases—a small improvement. We could then keep scaling up prevention programmes until it no longer improved things. In this example, with a budget of US$10 m the optimal split is US$5 m on treatment and US$5 m on prevention. Further shifts towards prevention would result in too little being spent on treatment, so the total number of cases would increase again from 1900 to 2100. Optimization algorithms automate this search process and often also include features to improve the speed and performance of the search.

## Optimally allocating health resources in practice

Constrained optimization methods have been in existence for many decades, but they are still very much the exception rather than the rule for determining how resources are allocated in health (Crown *et al.*, 2018). There are several reasons for this. Firstly, the data requirements for running a constrained optimization analysis can be extensive. Secondly, the technical knowledge required to set up a constrained optimization analysis can present a significant barrier. Thirdly, constrained optimization needs to be embedded within a broader decision-making framework. We address each of these points in the following sections.

### Data requirements

We saw in the previous section that evaluating the best allocation of resources involves understanding the potential outcomes under each feasible allocation. This either requires substantial data gathering (which may be impractical when there are more than a handful of feasible options) or constructing a model to estimate outcomes.

### Software and modelling support

Historically, setting up a constrained optimization analysis has required a substantial amount of coding and university-level mathematics. In recent years this has been evolving, however. The technological revolution of the past few decades has meant that people interact with extremely complex and technically involved algorithms on an everyday basis, but without requiring any knowledge about them because the interfaces for accessing these algorithms are so simple. Similarly, a number of software applications have been helping to put optimization algorithms into the hands of health-care decision-makers. For some optimization problems, the software available has already been commercialized and/or widely adopted (e.g. supply chain management software, various clinical management tools and roster optimization software). For other kinds of problems, software capacity has been increasing steadily but is yet to be widely adopted; e.g., although several tools exist to help optimize the location of health-care facilities (e.g. OptiDX, ArcGIS and AccessMod), actual decisions are often made by town planners or due to political reasons rather than with rigorous analysis. For the allocation of health-care budgets, our group developed the Optima suite of decision support tools, which includes web-based applications for optimizing budgets for HIV (Stuart *et al*., 2018; Kerr, *et al*., 2015), tuberculosis (Goscé *et al*., 2021) and nutrition (Pearson *et al*., 2018).

### Constrained optimization within decision processes

Decisions within health care are often made without explicit consideration of budget constraints, in which case cost-effectiveness analyses are an effective means of decision support; in this sense, constrained optimization methods can be considered as a useful complement to CEA methods (Wilson and Görgens, 2020). Both CEA and constrained optimization methods can be used to inform decisions within the context of a more holistic health system review, which may also assess factors such as purchasing/procurement arrangements, supply chain management, the funding landscape, equipment allocation and use and overall patient satisfaction, among others (Crown *et al.*, 2018; Verguet *et al*., 2021; Karsu and Morton, 2021).

## Conclusions

Constrained optimization methods provide a structured framework for answering a very broad class of problems: namely, how to best achieve a particular outcome, given a set of constraints. There are many health resource allocation problems where constrained optimization methods can help. If the problem consists of a goal, a set of possible strategies for attaining this goal and a set of constraints that must be fulfilled, then constrained optimization may be an appropriate method. If it is, then the next steps are to determine the expected outcome from each possible strategy and then to use constrained optimization algorithms to search among all possible strategies to find the one that leads to the best possible outcome.

Although all countries in the world have ostensibly committed to the United Nation’s Sustainable Development Goal of providing UHC, for many countries this was always going to be a difficult target to hit and has become even more difficult in the wake of the COVID-19 pandemic (Verguet *et al*., 2021). Moving beyond the immediate crisis presented by COVID-19, the detrimental impacts of the pandemic on countries’ health budgets, development assistance budgets and overall prosperity mean that it will be more critical than ever to identify methods for achieving the best outcomes possible with constrained means. The focus of this paper was on the ‘how’ of constrained optimization methods, but we also presented arguments for why such methods are useful.

Although there has been a proliferation of software for readily accessing constrained optimization methods, as well as a rapid escalation in the quantities and granularity of health data that are routinely collected (Murdoch and Allan, 2013), there are still barriers to using constrained optimization methods in practice. These barriers can partly be ascribed to the limitations of such methods, including their reliance on model-based estimates of the relationship between policies and health outcomes. Unfortunately, the limitations of constrained optimization methods can be particularly dangerous when they are hidden behind user-friendly interfaces, which often obscure the underlying processes and assumptions. In that sense, the increased availability of software for running constrained optimizations is a double-edged sword, making it both easier to apply such methods and more likely that they will be misapplied.

In this paper, we have tried to outline the steps for undertaking a constrained optimization analysis as simply as possible, as well as presenting arguments for why such an analysis is useful. With greater understanding of the methods, we hope that they might be applied more widely—and, even more crucially, accurately—over the years to come. Interested readers are referred to the excellent papers by the ISPOR Task Force (Crown *et al.*, 2017; Crown *et al.*, 2018) for examples of specific constrained optimization problems in health.

## Data availability

No new data were generated or analysed in support of this research.

## Reflexivity statement

The authors include four females and three males, with specializations in health policy, resource allocation, implementation, modelling, data science and epidemiology. All seven authors have extensive experience conducting allocative efficiency studies in close collaboration with partners from across Africa, Asia and Eastern Europe.
